# Molecular testing for *BRAF* mutations to inform melanoma treatment decisions: a move toward precision medicine

**DOI:** 10.1038/modpathol.2017.104

**Published:** 2017-11-17

**Authors:** Liang Cheng, Antonio Lopez-Beltran, Francesco Massari, Gregory T MacLennan, Rodolfo Montironi

**Affiliations:** 1 Department of Pathology and Laboratory Medicine, Indiana University School of Medicine, Indianapolis, IN, USA; 2 Unit of Anatomical Pathology, Faculty of Medicine, Cordoba University, Cordoba, Spain; 3 The Champalimaud Clinical Center, Lisbon, Portugal; 4 Division of Oncology, S. Orsola-Malpighi Hospital, Bologna, Italy; 5 Department of Pathology, Case Western Reserve University and University Hospitals Cleveland Medical Center, Cleveland, OH, USA; 6 Institute of Pathological Anatomy and Histopathology, School of Medicine, Polytechnic University of the Marche Region (Ancona), United Hospitals, Ancona, Italy

## Abstract

Approximately one-half of advanced (unresectable or metastatic) melanomas harbor a mutation in the *BRAF* gene, with V600E being the most common mutation. Targeted therapy with BRAF and MEK inhibitors is associated with significant long-term treatment benefit in patients with *BRAF* V600-mutated melanoma. Therefore, molecular testing for *BRAF* mutations is a priority in determining the course of therapy. A literature search was performed using MEDLINE/PubMed and scientific congress databases using the terms ‘BRAF,’ ‘mutation,’ and ‘cancer/tumor.’ These results were filtered to include manuscripts that focused on diagnostic tests for determining *BRAF* mutation status. Numerous *BRAF* testing methods were identified, including DNA-based companion diagnostic tests and DNA- and protein-based laboratory-developed tests. Herein we review the characteristics of each method and highlight the strengths and weaknesses that should be considered before use and when interpreting results for each patient. Molecular profiling has shown that mutation load increases with melanoma tumor progression and that unique patterns of genetic changes and evolutionary trajectories for different melanoma subtypes can occur. Discordance in the *BRAF* mutational status between primary and metastatic lesions, as well as intratumoral heterogeneity, is known to occur. Additionally, the development of acquired resistance to combination BRAF and MEK inhibitor therapy is still a formidable obstacle. Therefore, tumor heterogeneity and the development of acquired resistance have important implications for molecular testing and ultimately the treatment of patients with advanced-stage melanoma. Overall, this information may help community oncologists more accurately and effectively interpret results of diagnostic tests within the context of recent data characterizing melanoma tumor progression.

Melanoma represents a significant and increasing public health burden and an ongoing area of unmet need in oncology. Although melanoma accounts for only 1% of diagnosed skin cancers, it is the cause of most skin cancer-related deaths.^1^ In contrast to the incidence of other common cancer types, the incidence of melanoma is increasing.^2^ In the US, 87 110 new cases of melanoma and 9730 melanoma-related deaths are estimated in 2017.^2^ Until recently, patients with advanced melanoma had few effective treatment options; historically, response rates to conventional chemotherapy and immunomodulation therapy (interleukin 2 or interferon gamma) have been ~5–19%.^3, 4, 5, 6^ New therapeutic options include treatments targeted specifically to genetic mutations in patients’ tumors as well as immune checkpoint inhibitors.^7, 8, 9, 10, 11, 12^

Many different methods for *BRAF* testing are currently being used in the United States including US Food and Drug Administration (FDA)-approved companion diagnostic tests and various laboratory-developed tests.^13, 14, 15^ Information on FDA-approved tests for the detection of *BRAF* V600 mutations in melanoma is available at http://www.fda.gov/companiondiagnostics.^16^ Each *BRAF* testing method has its own unique strengths, weaknesses, and challenges that should be considered before using it to test a patient’s tumor sample.^13^ This review will discuss some of the currently available tests for determining *BRAF* mutation status in patients with advanced melanoma, focusing on considerations and approaches for accurate and effective testing in the community oncology setting.

## *BRAF-*mutant metastatic melanoma: an overview

A recent whole-genome sequencing study analyzing mutation frequencies across several cancers identified melanoma as the most frequently mutated tumor type;^17^ significant variation in the mutation profiles for different types of melanoma (eg, cutaneous, acral) has also been demonstrated by whole-genome sequencing.^18^ The majority of melanomas have mutations associated with the mitogen-activated protein kinase (MAPK) pathway, an important signal transduction pathway involved in cell growth, proliferation, and survival.^19^ Oncogenic activation of the MAPK pathway can occur via multiple mechanisms, the most common of which in melanoma is constitutive activation of the BRAF kinase via mutation, which occurs in ~40–60% of cases. The second most common MAPK pathway aberration in melanoma is mutated *NRAS*, occurring in ~15–30% of cases.^20, 21, 22, 23, 24, 25^

**Figure 1:**
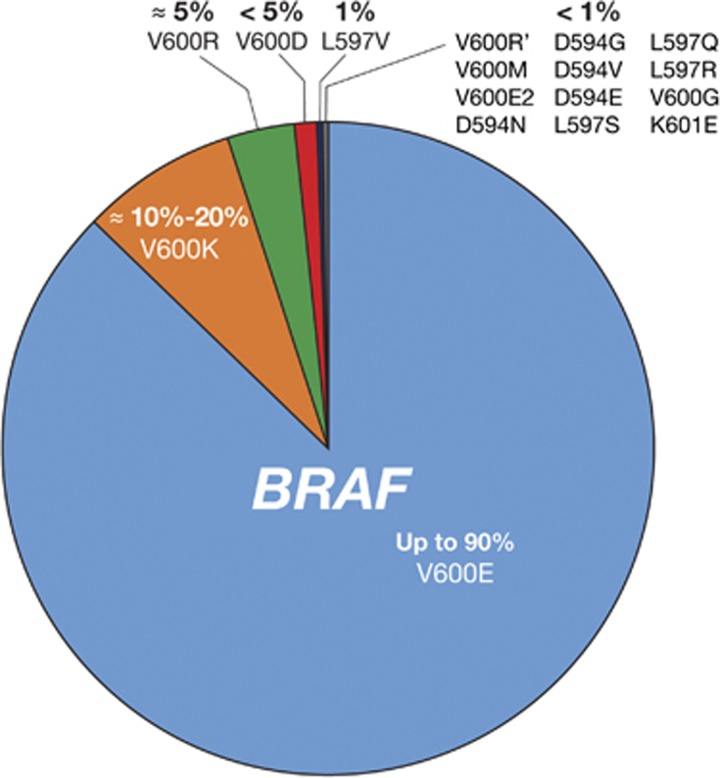
*BRAF* mutation types in melanoma. Estimated incidence of *BRAF* mutation frequencies in patients with melanoma is shown.^26^

*BRAF* encodes a cytoplasmic serine–threonine kinase. More than 97% of *BRAF* mutations are located in codon 600 of the *BRAF* gene.^13^ The most common mutation (in up to 90% of cases) is the result of a transversion of T to A at nucleotide 1799 (T1799A), which results in a substitution of valine (V) for glutamic acid (E) at position 600.^26^ Less common are substitutions of V for lysine (V600K (~8–20%)), arginine (V600R (1%)), leucine (V600M (0.3%)), and aspartic acid (V600D (0.1%)).^13, 24^ Non-V600 mutations (eg, K601E, D594N) are also known to occur in a low percentage of patients.^13, 27^ Types and frequencies of *BRAF* mutations are summarized in Figure 1 and Table 1.^26, 27^

*BRAF*-mutated melanoma tends to exhibit distinctive clinical features and is characterized by more aggressive biological behavior than *BRAF* wild-type (WT) melanoma. Compared with patients with *BRAF* WT melanoma, those with *BRAF*-mutated melanoma are more often younger and have tumors with superficial spreading or nodular histology and/or in anatomical regions without chronic sun damage.^24^ Furthermore, *BRAF*-mutant tumors are more likely to metastasize to the brain than *BRAF* WT tumors.^25^ *BRAF*-mutated melanoma has also been linked to shorter overall survival in patients with stage IV cancer than in those with *BRAF* WT disease.^24, 25, 28^ Given these features and the available effective therapies, it is crucial to quickly determine whether patients with melanoma have tumors harboring the *BRAF* mutation in order to select the optimal treatment.

The discovery that many melanomas harbor *BRAF* mutations^29^ led to the development of vemurafenib and dabrafenib, selective inhibitors of the BRAF V600-mutated kinase, and trametinib and cobimetinib, inhibitors of the downstream MEK kinase. Combination therapy with BRAF and MEK inhibitors (eg, dabrafenib plus trametinib, vemurafenib plus cobimetinib) has resulted in dramatic improvements in overall survival and progression-free survival rates in patients with *BRAF* V600-mutant advanced melanoma.^30, 31, 32, 33, 34, 35^ Three-year landmark analysis results have been presented from two randomized, phase 3 studies evaluating dabrafenib plus trametinib *vs* dabrafenib plus placebo (COMBI-d; NCT01584648) or single-agent vemurafenib (COMBI-v; NCT01597908) in patients with *BRAF* V600-mutant advanced melanoma.^36, 37^ The 3-year overall survival and progression-free survival rates were 44 and 22%, respectively, with dabrafenib plus trametinib *vs* 32% and 12%, respectively, with dabrafenib plus placebo in COMBI-d. In COMBI-v, overall survival and progression-free survival rates, respectively, at 3 years were 45 and 24% with dabrafenib plus trametinib *vs* 31 and 10% with vemurafenib. The safety profile of long-term combination therapy was similar to that in prior reports, with no additional safety signals observed. Three-year landmark data are also available from the phase 3 coBRIM study (NCT01689519), which evaluated cobimetinib plus vemurafenib *vs* placebo plus vemurafenib in patients with advanced *BRAF* V600-mutant melanoma; reported 3-year overall survival rates were 37.4 and 31.5%, respectively.^38^

Currently, *BRAF* mutation status is the only biomarker that predicts a therapeutic response in advanced melanoma.^39, 40^ Molecular testing for *BRAF* mutations in patients with advanced melanoma has become a standard for determining the course of therapy, and testing is recommended by the current National Comprehensive Cancer Network (NCCN) and European Society for Medical Oncology guidelines for melanoma.^40, 41, 42^ However, it is important to note that the NCCN Clinical Practice Guidelines in Oncology (2017) do not recommend testing the primary cutaneous melanoma for the *BRAF* mutation unless required to guide systemic therapy.^40^ BRAF inhibitors are approved only for use in patients with a *BRAF* V600 mutation detected by an FDA-approved test.^43, 44^ Patients with *BRAF* WT tumors may experience tumor promotion if treated with a BRAF inhibitor due to the paradoxical activity of the MAPK pathway in WT cells.^43, 44^ Per NCCN guidelines, first-line systemic treatment options for patients with metastatic or unresectable melanoma include immunotherapy (ie, anti-programmed death 1 (PD-1) receptor monotherapy (pembrolizumab or nivolumab) or nivolumab combined with the cytotoxic T-lymphocyte associated antigen 4 (CTLA4)-targeted antibody ipilimumab), targeted therapy (combination therapy with dabrafenib and trametinib or vemurafenib and cobimetinib) for patients with a *BRAF* V600 mutation, and enrollment in a clinical trial.^40^ In patients with a *BRAF* mutation, targeted therapy is preferred when an early response is clinically needed; however, the optimal sequence of BRAF inhibitors and immunotherapy is still under investigation. Because combination therapy with BRAF and MEK inhibitors has been shown to have the potential for significant long-term treatment benefit, identifying a patient’s *BRAF* mutation status should be a priority for the clinician.

## Evolution from benign to malignant melanoma: primary and secondary mutations

Although the *BRAF* V600 mutation is an oncogenic driver mutation involved in cellular proliferation, it is considered insufficient to induce melanoma in the absence of other cytogenetic abnormalities. It has been known for some time that *BRAF* V600 mutations are frequently found in benign and dysplastic melanocytic nevi, indicating that additional cellular changes are needed for transformation.^45^ These changes—including loss of tumor suppressors, activation of the *TERT* promoter, inactivation of genes involved in DNA repair, or activation of other protein kinases and signaling cascades^46^—in essence release the ‘brakes,’ allowing cellular proliferation to occur.^46, 47^ Common genes in which loss-of-function mutations can occur are *CDKN2A*, *PTEN*, and *BAP1.* Mutation of *CDKN2A*, for example, removes a key element of the p53 tumor suppressor pathway.^48^ Dysfunction of this pathway eliminates a mechanism that would normally consign precancerous cells with preexisting germline mutations or local, somatic mutations to apoptosis or senescence. Thus, cells that have a driver mutation combined with the loss of a tumor suppressor are primed to undergo transformation and develop into melanoma.

## Determining genetic heterogeneity of melanoma

**Figure 2:**
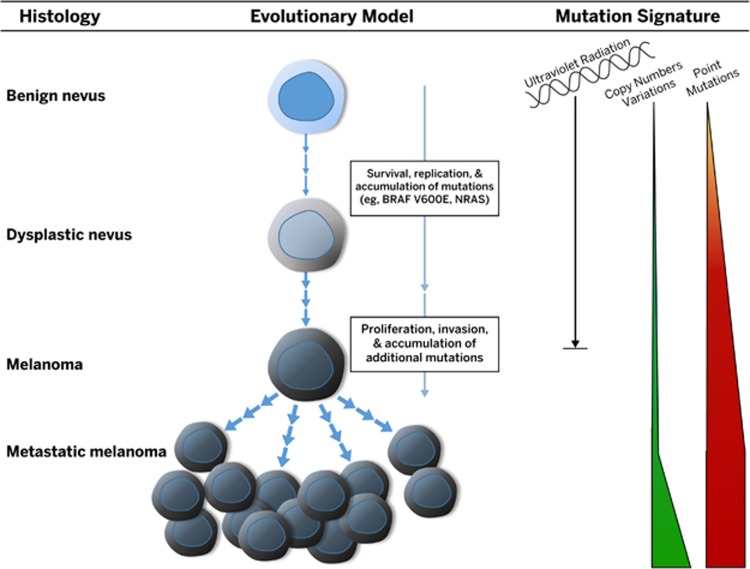
Genomic evolution of melanoma. Genetic alterations accumulate during melanoma progression, from benign lesions that carry the *BRAF* V600E mutation to intermediate lesions that have accumulated more genetic mutations to invasive melanoma that has acquired pathogenic mutations that confer the ability to metastasize. As melanoma progresses, distinct evolutionary trajectories for different melanoma subtypes develop.

Data have shown that the molecular profile of a tumor changes over time due to various selection pressures.^46, 47^ Tumor heterogeneity in advanced-stage melanoma has important implications for molecular testing and thus treatment. Shain *et al.* examined 37 primary melanocytic neoplasms and their adjacent precursor lesions, ultimately microdissecting and sequencing 150 distinct areas^46^ (Figure 2). Interestingly, tumors that lacked malignant behavior harbored only *BRAF* V600E mutations. The mutation load increased with progression, and unique patterns of genetic changes, termed ‘evolutionary trajectories,’ for different melanoma subtypes were observed. Intermediate lesions were enriched for *NRAS* mutations and additional driver mutations. Progression was marked by the appearance of *TERT* promoter mutations and an increasing burden of *NRAS* mutations in intermediate lesions. This was followed by inactivation of *CDKN2A* or mutations in SWI/SNF chromatin remodeling genes in invasive melanomas and loss of *PTEN* and *TP53* in advanced melanoma. In rare cases, lesions had both *BRAF* V600E and *NRAS* mutations identified. Although it is thought that *BRAF* and *NRAS* mutations are mutually exclusive, this study by Shain *et al.* and another recent study by Chiappetta *et al.* showed that these mutations can both be present, most likely due to clonal heterogeneity within the tumor.^46, 49^

Researchers have evaluated the discordance in *BRAF* mutation status between primary and matched metastatic lesions in relation to the potential implications for therapeutic success and treatment decision-making. Heinzerling *et al.* evaluated *BRAF* mutation heterogeneity between tumor samples isolated from the primary tumor and those from metastases from patients with melanoma.^50^ In this study, the tumors were not under the selection pressure of a BRAF inhibitor because tumor samples were collected prior to the availability of BRAF inhibitor therapy. A total of 53 of the 187 patients with melanoma had multiple tumor samples available for analysis. Ten of 53 patients (19%) had discordant tumor samples that harbored both *BRAF* WT and mutation-positive metastases. *BRAF* mutations were as likely to first appear in metastases derived from WT tumors as they were to be found in the primary tumor that subsequently had WT metastases. Additionally, similar to results reported earlier by Shain *et al*,^46^ considerable intratumoral heterogeneity was found using immunohistochemistry (IHC) staining for mutated *BRAF*, suggesting the appearance of *de novo* *BRAF* mutations in the metastases of some patients. The investigators speculated that *BRAF* mutations may arise spontaneously in the secondary tumor from a *BRAF* WT primary tumor. Alternatively, genetically heterogeneous metastatic tumors could derive from *BRAF* mutation-positive or *BRAF* WT primary tumor cells, leading to intrapatient discordance. It is also possible that these results may have been a result of sampling or the detection method used.^50^

Yancovitz *et al.* used mutation-specific real-time polymerase chain reaction (RT-PCR) to examine intra- and inter-tumoral heterogeneity of the *BRAF* mutation.^51^ To determine intratumoral heterogeneity, laser microdissection of three to five sites from nine primary melanomas was performed and showed variability in the percentages of *BRAF-*mutant cells across the tumor sites. Samples were classified as unlikely to be heterogeneous (three patients), likely to be heterogeneous (two patients), or having marked heterogeneity (four patients). To determine intertumoral heterogeneity, samples from 18 patients with matched primary and secondary tumors and 19 patients with tumors from more than one metastatic site were analyzed for concordance in *BRAF* mutations. Discordance was observed between primary and metastatic tumors in 8 of 18 patients (44%) and between metastatic tumors in 5 of 19 patients (26%). The investigators suggested that primary melanomas may contain mixed populations of *BRAF-*mutant and *BRAF* WT cells, both able to metastasize, and that resistance to targeted therapies may be mediated by these genetically distinct tumor subclones.^51^ Data in support of this idea were reported in a study by Colombino *et al.*, who analyzed 291 samples from 132 patients with melanoma.^20^ Although *BRAF* mutations were detected in 43% of primary tumors, the rate of *BRAF* mutations in metastases was 5% greater (48%), consistent with accumulation of *de novo* genetic abnormalities as melanoma develops. Interestingly, *BRAF* mutation concordance varied by metastasis site: concordance with the primary tumor was highest in lymph node and visceral metastases (93 and 96%) and lowest in skin and brain metastases (75 and 80%).^20^

Bradish *et al.* evaluated the concordance in *BRAF* mutational status between primary and matched metastatic lesions collected from 25 patients with metastatic melanoma.^52^ Six of these patients also had multiple metastatic lesions available for testing. None had received treatment with a BRAF inhibitor. Using an RT-PCR kit able to detect five different somatic *BRAF* V600 mutations—including the most common V600E and less common V600K, V600D, and V600R—intertumoral discordance was detected in the tumors of four patients (16%). Of these four patients, two had *BRAF* WT primary lesions with *BRAF* V600-mutant metastatic lesions and the other two had *BRAF* V600-mutant primary lesions with *BRAF* WT metastatic lesions. Additionally, the discordance rate was higher in patients with multiple metastases (two of six (33%)) than in patients with a single metastasis (two of 19 (11%)), indicating that increasing the number of lesions tested increases the likelihood of a discordant finding.

Together, these studies indicate that detection of a negative *BRAF* mutation in the primary tumor may not necessarily reflect the *BRAF* mutation status of metastases. Therefore, clinicians should consider repeat testing of the primary tumor and recent metastatic site(s) to determine the *BRAF* mutation status.^52, 53, 54^

## Resistance to BRAF and MEK inhibitor therapy

MAPK pathway inhibitors are an option for patients with *BRAF* V600-mutant advanced melanoma.^26, 40, 55^ The introduction of BRAF inhibitor therapy was an important step forward in the treatment of *BRAF-*mutated melanoma. Yet up to one-half of patients with *BRAF-*mutated tumors did not respond to treatment in previous studies; in those who did respond, acquired resistance generally developed at a median of 6–7 months with single-agent BRAF inhibitors.^56, 57^ Combination treatment with BRAF and MEK inhibitors has been shown to significantly increase the proportion of patients with an objective response while also significantly delaying the development of acquired resistance.^33, 36, 37, 58, 59^ Therefore, combination therapy (dabrafenib plus trametinib or vemurafenib plus cobimetinib) is now considered a standard of care in patients with *BRAF*-mutated advanced melanoma.^40^

Mechanisms of *de novo* and acquired resistance to BRAF inhibitor monotherapy were recently described in a comprehensive review article by Manzano *et al* ^60^ *In vitro* and *in vivo* models have identified primary resistance mechanisms that drive proliferation despite inhibition of *BRAF* V600 mutations, including alterations in *RAC1,* loss of *PTEN*, changes in cyclin-dependent kinase 4 and cyclin D1, and stromal secretion of hepatocyte growth factor activating c-MET.^60^ Several mechanisms of acquired resistance have also been identified, including the selection of subclonal populations that do not express mutated *BRAF* (as previously discussed) or populations that have alterations or mutations in components of the MAPK (eg, *NRAS* mutations, upregulation of tyrosine kinase receptors) or PI3K/AKT pathways (eg, loss of *PTEN*) or changes in downstream targets (eg, overexpression of transcription factors). As with *de novo* resistance, these mechanisms can drive proliferation.^61^

Although significant improvement in survival has been achieved with combination BRAF and MEK inhibition, *de novo* and acquired resistance continue to be formidable obstacles.^59^ Patients with disease progression on BRAF inhibitor therapy who subsequently received combination BRAF and MEK inhibitor treatment had an overall response rate of 10–15%, indicating limited clinical benefit from combination therapy once resistance had developed.^62, 63^ Long *et al.* evaluated resistance mechanisms in 20 *BRAF* V600E-mutant tumor samples from 10 patients with early resistance (≤6 months of dabrafenib plus trametinib prior to progression). MAPK pathway reactivation, indicating an ‘addiction’ to the MAPK pathway, was observed in 9 of 10 tumors (90%). Resistance mechanisms included *BRAF* amplification as well as mutations in *MEK1/2* and *NRAS*.^64^ In this and a second study by Rizos *et al*, the rate of MAPK pathway reactivation was higher than that previously observed in patients who progressed on a BRAF inhibitor alone (50%).^64, 65^

*In vitro* studies evaluating the pathophysiology of acquired resistance to combination therapy have shown that resistant melanoma cell lines exhibit a much greater degree of drug addiction than cell lines resistant to BRAF inhibitors alone.^66^ Similar to what has been observed in tumors of patients who progressed on a single BRAF inhibitor and were then treated with BRAF and MEK inhibitors, BRAF inhibitor-resistant cell lines demonstrated the ability to rapidly adapt to combination therapy. This is thought to be due to pre-existing resistance mechanisms. The researchers suggested that intermittent drug dosing may be a strategy to delay the development of acquired resistance, and further studies are currently testing this hypothesis.^66^

A reduced ability to mount an antitumor immune response has also been observed in patients who progressed on BRAF inhibitor monotherapy and then received the combination of BRAF and MEK inhibitor therapy.^63^ Chen *et al* evaluated 14 tumor samples from a cohort of 23 patients refractory to BRAF inhibitor therapy who were switched to combination therapy. Prior to being treated with combination therapy, the tumors showed a relatively low number of tumor-infiltrating CD8-positive T cells and limited expression of programmed death-ligand 1 (CD274; previously named PD-L1). When patients were switched to combination therapy, an increase in immune cell infiltrate into the tumor was observed in one of nine evaluable patients who achieved stable disease.^63^

There is also great interest in combining BRAF and MEK inhibitor therapy with immune checkpoint inhibitor therapy.^67^ Preliminary animal models evaluating the combination of MAPK pathway inhibitors with checkpoint inhibitors suggest that the combination may result in greater clinical benefit than what can be achieved using either agent alone. Working with tumor-bearing mice, Ebert *et al* showed that MEK inhibition stimulated the infiltration of antigen-specific CD8-positive T cells and prevented upregulation of programmed cell death protein 1 (PDCD1). Furthermore, combining MEK inhibition with anti-PDL1 therapy resulted in synergistic antitumor activity.^68^ Another animal study evaluated BRAF plus MEK inhibition combined with anti-PDCD1 therapy and demonstrated superior antitumor activity with the triple combination over combined BRAF plus MEK or PDCD1 inhibition alone.^69^ An ongoing clinical study (KEYNOTE-022; NCT02130466) is evaluating dabrafenib plus trametinib plus pembrolizumab in patients with *BRAF-*mutant advanced melanoma.^67^ Another ongoing study is evaluating sequencing of combination targeted therapy and checkpoint inhibitors (EA6134; NCT02224781).

## *BRAF* V600 mutation diagnostic tests

### Obtaining and Processing Materials for BRAF Testing

**Figure 3:**
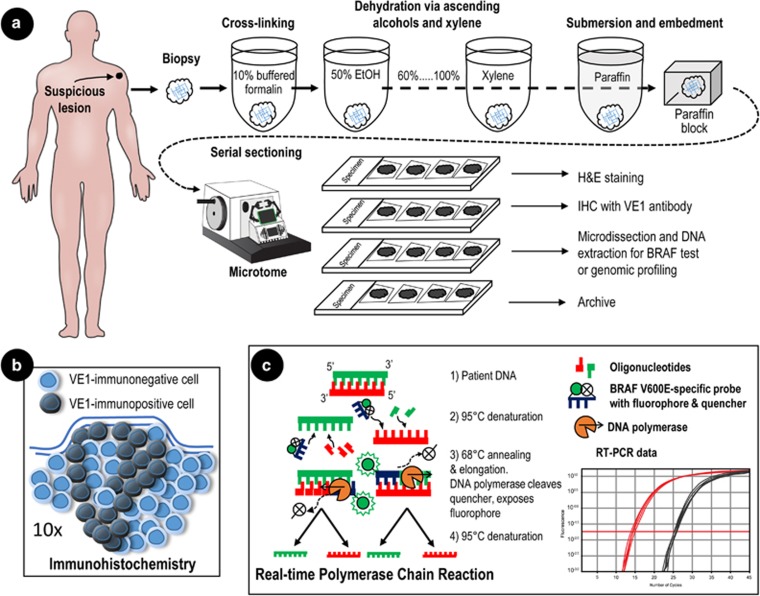
Processing of lesions for pathological analysis of *BRAF* V600 mutation. (**a**) The suspicious lesion is biopsied and prepared for formalin-fixed, paraffin-embedded tissue. The tissue sample is immersed in 10% buffered formalin for 4–6 h, then immersed in a series of increasingly concentrated ethanol to extract water. The final dehydration occurs in xylene, after which the tissue is immersed in a series of increasingly concentrated molten paraffin. Finally, the sample is embedded in a block of paraffin suitable for serial sectioning. Serial sectioning produces ‘ribbons’ of tissue sections that can be directly mounted onto slides, deparaffinized, and then stained. Every other section is stained, typically with hematoxylin and eosin, to identify tumor margins while unstained alternate sections are used for immunohistochemistry (IHC) or to obtain DNA for genetic analyses. In contrast to the process for surgical specimens, fine-needle aspirates are taken and transferred directly from the syringe to a slide, where the material is smeared across its surface, air dried, and fixed. (**b**) IHC reveals cellular expression of the BRAF kinase in *BRAF* mutation-positive (dark gray) cells *in situ*. (**c**) RT-PCR uses a fluorescent-labeled target-specific probe allowing amplification of the target sequence and real-time quantification of PCR products. EtOH, ethyl alcohol.

After a preliminary diagnosis of stage III or IV metastatic melanoma, the lesion(s) should be evaluated for *BRAF* mutational status.^40^ Cutaneous lesions suspected of being melanoma are typically resected with a wide margin, providing the entire lesion and its margins for analysis. Radiologically detected lesions within internal organs are sampled by core-needle and/or fine-needle aspiration biopsy. The postsurgical processing of surgical resections *vs* fine-needle aspirates is slightly different. Biopsy specimens are usually processed by formaldehyde fixation and paraffin embedding in blocks for subsequent IHC or DNA extraction for sequencing^70, 71^ (Figure 3).

Alternatively, a blood sample (‘liquid biopsy’) may be assayed for circulating tumor DNA (ctDNA).^72, 73^ ctDNA is composed of small fragments of nucleic acids that are found in the circulation but are not associated with any cell or cell fragment. Although the mechanism by which ctDNA is released into the blood is unclear, its abundance seems to reflect the biological aggressiveness of the tumor.^74, 75, 76, 77^ Despite being an important innovation in cancer diagnostics, ctDNA detection has limitations compared with a tissue-based biopsy. Currently, ctDNA analysis is less sensitive than tissue biopsy. Santiago-Walker *et al* estimated the sensitivity of ctDNA testing for *BRAF* V600E to be 76% relative to standard PCR using a direct tumor biopsy. This was determined by calculating the concordance in *BRAF* V600E-positive mutation status between tissue biopsies and ctDNA (*N*=661).^78^ Furthermore, because blood lacks anatomical specificity, the presence of *BRAF-*mutant DNA in blood does not indicate the number of tumors or the tumor origin. Additional diagnostic tests are still required to identify the tumor site(s), which may add to the overall expense, unlike conducting a standard biopsy and mutational test.

Despite these limitations, ctDNA can provide useful information for treatment monitoring. The correlation between ctDNA abundance and disease stage has been examined in patients with melanoma. The quantity of *BRAF* V600E mutation-specific DNA has been shown to be positively correlated to tumor burden and disease stage.^78, 79, 80^ Accordingly, patients with *BRAF*-positive ctDNA had significantly shorter progression-free and overall survival than patients without detectable *BRAF*-positive ctDNA.^78^ The quantity of *BRAF* V600E-positive ctDNA was also shown to be greater in patients with more metastatic sites.^81^

### BRAF Testing: Companion Diagnostic and Laboratory-Developed Tests

BRAF genotype tests are classified as either companion diagnostic or laboratory developed, and US and European regulatory agencies have issued guidelines on their use. The European Medicines Agency summary of product characteristics for vemurafenib states that before starting treatment, a validated test is required to identify *BRAF* mutation status but does not specify which test should be used. It does state that the cobas 4800 BRAF V600 Mutation Test (Roche Molecular Systems, Inc) was used to identify *BRAF* mutation-positive patients in the clinical trial on which marketing approval was based.^82^ In the US, companion diagnostic tests are often codeveloped with a drug or drug class and are recommended and approved by the FDA for informing the safe and effective use of its corresponding therapeutic product.^83^ Companion diagnostic tests undergo rigorous testing and are reviewed by the FDA and the US Centers for Medicare and Medicaid Services to ensure analytical and clinical validity. In contrast, laboratory-developed tests are typically designed, developed, and used by a single institution, such as a hospital or research laboratory.^84, 85^ Unlike companion diagnostic tests, laboratory-developed tests are not linked to a particular drug or drug class. Two companion diagnostic tests are commercially available for detection of *BRAF* mutations: the cobas 4800 BRAF V600 Mutation Test for vemurafenib plus cobimetinib and the THxID-BRAF kit (bioMérieux, Inc) for dabrafenib plus trametinib.^16^ In addition, laboratory-developed tests may be used to determine a patient’s *BRAF* mutation status, provided that the tests are reviewed and tested by the US Centers for Medicare and Medicaid Services and used in a Clinical Laboratory Improvement Amendments (CLIA)-certified laboratory.^84, 86^ In a recently published survey of laboratories participating in the College of American Pathologists proficiency testing program for molecular analysis of *BRAF* mutations, most evaluable reports (100 of 107 (94%)) included only laboratory-developed procedures (the remaining 7 included a companion diagnostic assay).^87^ Although the extent of regulatory oversight of laboratory-developed tests has been minimal, the FDA has developed more robust guidance as the complexity of tests has increased. The FDA issued draft guidance on the oversight of laboratory-developed tests in 2014 and issued a discussion paper in early 2017.^84, 88, 89^

### Current and Developing Tests

Several *BRAF* mutation tests are currently in use; they are classified as DNA based or antibody based.^90^ DNA-based tests include those that use PCR to selectively amplify the mutant *BRAF* gene and others that directly determine the nucleotide sequence of the *BRAF* gene.^26, 90^ Of note, PCR and other DNA-based tests reveal the presence (or absence) of the gene but do not indicate whether the gene is being transcribed and translated into a protein.^70, 71^

Currently, the only available antibody-based test for mutant BRAF protein uses the monoclonal antibody VE1 to detect expression in tumor samples using IHC.^26, 90, 91^ This approach is effective in identifying a qualitative change (ie, presence or absence of the protein) but is less accurate in quantitating changes in expression than other antibody-based assays, such as the enzyme-linked immunosorbent assay.^92^

Regardless of the technical approach used, every test can be characterized by its sensitivity, specificity, and limit of detection. Sensitivity is the ability of the test to correctly identify *BRAF* mutations with a low rate of false negatives. Specificity is the ability of the test to correctly identify *BRAF* mutations with a low rate of false positives. Limit of detection is the threshold at which a signal (eg, DNA harboring the *BRAF* mutation) can be distinguished from the background (eg, *BRAF* WT DNA).^93, 94^ Tests also vary in terms of their selectivity.^94, 95^ For example, some tests can detect only the V600E mutation, while others can detect V600E and other mutations, such as V600K.^14, 15, 91^ This is important because some agents (vemurafenib plus cobimetinib, trametinib, and dabrafenib plus trametinib) have been shown to be effective in patients with V600E/K mutations.^26, 31, 32, 33^

### PCR DNA-Based Tests

The cobas 4800 BRAF V600 mutation test and the THxID-BRAF kit both use RT-PCR to amplify and detect the mutant DNA sequence in a tumor sample. The major advantages of RT-PCR are faster performance, better reproducibility, and lower cost compared with traditional genomic sequencing methods.

The RT-PCR process is illustrated in Figure 3 and includes an example of a data readout from an RT-PCR assay. Briefly, the RT-PCR-based BRAF test uses one primer set to detect the *BRAF* mutation and another set to detect the WT sequence.^14, 45^ Each primer includes a distinct fluorophore and quencher. The quencher prevents the excitation/emission of light by the fluorophore as long as the primer is unbound to DNA. As the DNA polymerase incorporates the primer into a new DNA strand, it cleaves the quencher from the primer, freeing the fluorophore to emit narrow-bandwidth light that can be detected. As DNA is synthesized with each cycle, the quantity of light increases relative to the abundance of the DNA product, which is determined by the number of cycles necessary for the intensity of emitted light to cross an arbitrary threshold. The relative abundance of *BRAF* V600E is determined by comparing this threshold with the number of cycles necessary to detect WT *BRAF*.

It is recommended that patients undergo an FDA-approved test, such as the cobas 4800 BRAF V600 mutation test or THxID-BRAF kit, before starting treatment with vemurafenib plus cobimetinib or dabrafenib plus trametinib.^14, 15^ The cobas 4800 test has an analytical sensitivity of 95% for detecting the V600E mutation.^82^ However, the test shows limited cross-reactivity in detecting the V600D and K mutations, and the package insert states that the test does not detect non-V600E mutations reliably. The recommended DNA input is 125 ng total, since the test can detect the *BRAF* V600E mutation at ≥5% using the standard input of 125 ng/25 μl.^15^ The THxID-BRAF kit detects both V600E and K mutations. The kit has been validated for DNA input of 10 ng/μl to 350 ng/μl.^14^ In contrast to the cobas 4800 test, the THxID test has a high degree of sensitivity for both V600E and V600K (Table 2).

Although the RT-PCR-based cobas 4800 and THxID assays are the companion diagnostic tests for BRAF plus MEK inhibitor use, many treatment centers use laboratory-developed tests based on other DNA sequencing methods.^87, 90^ The goal of genomic sequencing in the context of BRAF testing is to identify any one of the nucleic acid substitutions associated with the mutant *BRAF* gene (Table 2). The advantage of genomic sequencing is that it is not specific for a certain mutation; therefore, any change in the sequence may be identified. Three main methods are currently being used: Sanger sequencing, pyrosequencing, and high-resolution melt analysis/real-time PCR.^87, 90, 96^ Next generation sequencing platform is less frequently used in clinical labs and is beyond the scope of this review.^90, 97^

#### Sanger sequencing

The Sanger sequencing method, generally considered to be the gold standard, was developed in the 1970s and was the basis for the method used by Venter *et al* in the Human Genome Project to generate the first draft human genome sequence.^98^ The Sanger method relies on incorporation of deoxynucleotide triphosphates (dNTPs; dATP, dCTP, dGTP, and dTTP) into a complementary strand of DNA that exactly matches a denatured target strand. The nucleotide sequence is detected by chain termination, which results in DNA strands of variable length. Chain termination occurs due to the incorporation of 2′,3′-dideoxynucleotide triphosphates (ddNTPs), which lack a 3′-OH and thus cannot form a bond with the next dNTP.^99^ Reactions with each of four ddNTPs (ddATP, ddCTP, ddGTP, and ddTTP) that are tagged with a radioactive phosphate moiety or fluorophore are run in parallel and resolved by electrophoresis. The length of each DNA product indicates where the chain terminated and which nucleotide was the last to be incorporated. Advantages of Sanger sequencing include reliability and availability and affordability of the reagents; the primary disadvantage is its relatively low sensitivity.

Jurkowska *et al* recently compared the reproducibility of Sanger sequencing with that of the cobas 4800 test. Among 236 formalin-fixed, paraffin-embedded tissue (FFPET) samples,^100^ Sanger sequencing identified the V600E mutation in 60.9% of samples and the cobas 4800 test identified it in 61%, with 95.2% agreement between the two tests. Sanger sequencing also detected 10 non-V600E mutations, while the cobas 4800 test detected 6. Six cases were unamplifiable with the standard PCR/sequencing method, five of which were identified as V600E positive by the cobas 4800 test.

Lopez-Rios *et al* also compared Sanger sequencing with the cobas 4800 test.^101^ The Sanger method produced invalid results in 8 of 116 samples *vs* 0 of 232 with the cobas 4800 test. The positive percent agreement with Sanger sequencing was 97.7% the negative percent agreement was 95.3%.

Qu *et al* performed a similar analysis and found lower sensitivity for the cobas 4800 test than for the Sanger method.^102^ In 275 FFPET samples, V600 mutations were identified in 35 and 43%, respectively. Compared with Sanger sequencing, the cobas 4800 test exhibited 80.5% sensitivity and 99.4% specificity. Of interest, 23 samples that were positive with Sanger sequencing were discordant with the cobas 4800 test. Based on these results, the authors suggested that Sanger sequencing should be used to test samples that are negative by the cobas 4800 test.

#### Pyrosequencing

In the early 2000s, multiple innovations in sequencing methods were introduced. One example is pyrosequencing. Like the Sanger method, pyrosequencing relies on DNA synthesis, but it detects the addition of new base pairs using an enzymatic reaction that ultimately produces light.^103^ The process starts when target DNA is mixed with one of four dNTPs. As the DNA polymerase incorporates the complementary dNTP (A to T and C to G) into the strand, a molecule of pyrophosphate is released. The presence of sulfurylase in the reaction converts pyrophosphate to adenosine triphosphate, which interacts with luciferase, producing light. Each flash of light can be detected with a charge coupled device camera, indicating the addition of specific dNTPs to the growing strand.^104^ As with the Sanger method, a separate reaction is required for each dNTP. However, four separate reactions can be conducted simultaneously, greatly speeding up the process.

Ihle *et al* used *BRAF*-specific pyrosequencing, high-resolution melt analysis, multigene next-generation sequencing, and IHC and compared these techniques with the Sanger method for sensitivity and specificity in detecting the *BRAF* mutation in FFPET samples.^13^ Pyrosequencing was found to have lower specificity than Sanger sequencing (90 *vs* 100%) but also a lower limit of detection. It detected mutant DNA in as few as 5% of copies compared with 6.6% with Sanger sequencing and 7% with RT-PCR (Cobas 4800).

As with the Sanger method, multiple biological steps are required for pyrosequencing. The process takes 2–4 days to complete, the run itself is 10 h, and additional time is required for bioinformatics analysis. A key advantage of pyrosequencing is that it has been adapted to a high-throughput platform, vastly increasing the amount of generated data.^103^ The disadvantages of pyrosequencing are high cost and increased complexity and time. A high-throughput pyrosequencing machine can cost >$600 000, and some reagents cost thousands of dollars.^103^ However, because of the longer reads made possible by pyrosequencing, the cost per base is lower than that for Sanger sequencing. In addition, sequencing the genome is unnecessary to determine the presence or absence of the *BRAF* V600E mutation, and the potential to detect multiple mutations with pyrosequencing has led to its increased use as a diagnostic tool.

### High-Resolution Melt Analysis/Real Time PCR

High-resolution melt is a sequencing technique that incorporates aspects of PCR and sequencing by DNA synthesis. High-resolution melt is a relatively simple sequencing technique, as it is based on the principle that double-stranded DNA denatures (ie, ‘melts’) at a temperature that varies as a function of its G/C content, length, and overall nucleotide sequence.^105^ The process normally begins with amplification of the target DNA to nanogram quantities using PCR.^105^

Intercalating dyes (eg, SYBR Green, LC Green, SYTO 9, and EvaGreen) are included in the reaction, binding to double-stranded DNA but not single-stranded DNA.^106^ Thus, fluorescence will initially be high because the sample is composed solely of newly synthesized, double-stranded DNA. While fluorescence is continually monitored, the PCR-amplified sample is heated through a range of temperatures. As the temperature gradually increases and the strand begins to denature, the fluorescence slowly decreases. Then, at a temperature characteristic of the DNA strand’s G/C content, length, and overall nucleotide sequence, the fluorescence falls rapidly, indicating the complete separation of DNA into single strands. The substitution of a single base can lead to a 1 °C difference in the melting point, a difference easily detected by the melting curve shape. Data are usually presented as the derivative of the melting curve, showing the change in fluorescence over the change in temperature. The sensitivity of the technique is directly related to temperature control. The RapidCycler II (BioFire Diagnostics, Inc (formerly known as Idaho Technology, Inc)) and LightCycler 480 (Roche Diagnostics) melts samples at 0.3 °C/sec, whereas the Rotor-Gene 6000 (Corbett Life Science, a QIAGEN company) controls temperature at 0.017 °C/sec. The slower melting time results in higher resolution and better performance; however, the tradeoff is speed of data acquisition.^105^ The RapidCycler can acquire data in 1–2 min, whereas the Rotor-Gene device requires 20 min.^105^

The advantages of using high-resolution melt over other techniques include low cost and increased sensitivity and convenience. The PCR step and high-resolution melt steps can occur in the same tube or capillary, lowering the possibility of contamination and reducing the likelihood of operator error. The LightCycler 480 and RapidCycler II both provide integrated PCR and high-resolution melt analysis in one machine. Additionally, the technique is not destructive, allowing additional analyses on the same material. A disadvantage of high-resolution melt is that it does not permit the direct identification of the nucleotide sequence. However, from a community clinician’s perspective, this may not disqualify it as a technique since it screens for the presence of the *BRAF* mutation, which is sufficient for deciding whether to administer BRAF inhibitor therapy.

A meta-analysis of 14 studies that included 1324 samples from patients with melanoma and other cancers examined the sensitivity of high-resolution melting in detecting *BRAF* mutation.^106^ Most of the samples were obtained from FFPET, although some were from fresh-frozen tissue. The pooled value of the sensitivity of the high-resolution melting curve analysis was high: 0.99 (range, 0.93–1.00). The pooled specificity of the high-resolution melting curve analysis was also 0.99 (range, 0.88–1.00).

Richter *et al* compared the *BRAF* mutational testing accuracy of four techniques—high-resolution melt, single-strand conformation analysis, Sanger sequencing, and RT-PCR—in a blinded test across two laboratories of >90 FFPET samples from melanoma patients. They found 100% concordance between the Sanger and high-resolution melt techniques.^96^ Together, these results indicate a high level of overall accuracy for high-resolution melt.

### Protein Based Analysis (immunohistochemistry)

Although molecular testing is currently considered to be the gold standard for the detection of *BRAF* mutations, the monoclonal antibody VE1, developed to specifically recognize a segment of the mutant BRAF V600E protein, is emerging as a reliable option.^107, 108^ Since BRAF has a role in intracellular signaling, BRAF staining is localized to the cytoplasm.^109^ There are both advantages and disadvantages to IHC detection of mutant BRAF.^96^ Advantages include visualization of BRAF V600E-mutant protein distribution at the single-cell level, semiquantitative readout of protein abundance, and high assay sensitivity and specificity. Because FFPET samples are usually available as part of a regular biopsy, testing by IHC should be relatively simple and inexpensive, and provide a fast turnaround on results. The main disadvantages of IHC include the possibility of false negatives due to high heterogeneity or low abundance of *BRAF* V600E and a lack of availability of antibodies for *BRAF* V600K or other variants. In addition, because IHC recognizes protein and not DNA, there may be discrepancies between IHC and DNA-based test results. Despite these drawbacks, reports have indicated that the sensitivity and selectivity of the VE1 antibody compare favorably with those of DNA-based tests.

Hugdahl *et al* used the VE1 antibody to assess the frequency of the mutant BRAF V600E protein in 248 nodular melanomas and investigate its prognostic value.^28^ They found BRAF V600E staining in 35% of the cases. Tumors with BRAF V600E staining were thicker and more likely to be ulcerated, and patients with these lesions had reduced survival. Despite these observations, BRAF V600E protein expression independently predicted reduced survival, separately, and distinctly from mitotic rate, presence of ulceration, and tumor thickness. The concordance between V600E staining and mutation status, as assessed by PCR, was 88%. In a comparison of Kaplan–Meier survival curves in patients grouped by BRAF protein expression (*n*=248) and those grouped by *BRAF* mutation status (*n*=191), the researchers found that expression of the mutant BRAF protein was a better prognostic indicator than presence of the mutant *BRAF* gene. However, caution should be taken when interpreting this finding, since the analysis based on BRAF protein expression included more patients than the analysis based on *BRAF* gene presence and thus had more statistical power.

Long *et al* compared the sensitivity and specificity of VE1 immunostaining with those of a DNA-based assay in 100 patients.^110^ VE1 immunostaining had a sensitivity of 97% (37 of 38 paired samples yielded identical results) and specificity of 98% (58 of 59) for detecting the presence of the *BRAF* V600E mutation. The authors noted that ‘…clinical use of the V600E BRAF antibody should be a valuable supplement to conventional mutation testing and allow V600E mutant metastatic melanoma patients to be triaged rapidly into appropriate treatment pathways.’ Further support for this conclusion was provided by a study by Pearlstein *et al*, who compared VE1 immunostaining results with pyrosequencing results in 76 patients with melanoma.^111^ A total of 27 patients (35%) had the V600E mutation, and another nine had an alternate mutation (V600K, V600R, or V600Q). Of these, there were five discordant cases in which pyrosequencing reported the presence of the V600E mutation but immunostaining was negative. Further analysis of these discordant findings showed that two of the five cases had a high percentage of V600E allele frequency while two others demonstrated a low allele frequency. The samples with a low allele frequency suggest the possibility of tumor heterogeneity. The final discordant case was reclassified as it was found to have a *BRAF* V600K rather than V600E mutation. Thus, the IHC test had a sensitivity of 85% and specificity of 100% compared with pyrosequencing.

Manfredi *et al* compared RT-PCR and high-resolution melt analysis with IHC.^112^ Of 189 samples, 88 (47%) tested positive for BRAF V600E mutation by IHC. The V600E-positive samples were analyzed by genotyping of 74 samples, which yielded interpretable results. Of these, only two samples were not confirmed by both genotyping methods. Of the 87 samples that were determined to be BRAF V600E negative by IHC, 76 (87%) were determined to be BRAF WT by molecular testing. Ten of the remaining samples were determined to be other BRAF V600 variants. Only one was determined to be a true false-negative, as it was determined to be BRAF V600E following molecular testing. Overall, this analysis demonstrated that IHC testing with VE1 had high sensitivity (98.6%) and specificity (97.7%) for IHC.^112^

Another challenge introduced by IHC staining is the potential for subjective differences in interpretation between pathologists. A study by Eriksson *et al* evaluated VE1-stained FFPE melanoma samples and compared the reported interpretations with the results of DNA sequencing.^109^ They asked three observers to judge the staining intensity of samples on a three-point semiquantitative scale. This method resulted in a sensitivity of 96.7% and specificity of 94.5% for IHC with VE1. The level of agreement between the observers was not reported. A 2014 study by Marin *et al* illustrated the potential challenges associated with subjectivity introduced with interpretation of IHC.^113^ In that study, seven pathologists, two of whom had been previously trained to interpret V600E staining, reviewed VE1 staining in samples from 67 patients. Concordance between the seven pathologists was achieved only 79.1% of the time. This study emphasizes that VE1 staining has some diagnostic limitations and highlights the importance of training. Overall, the authors of this study and others conclude that IHC can serve as an important first step in determination of BRAF V600E mutation status.^96^

## Conclusions

The introduction of combination BRAF and MEK inhibitor therapy has transformed treatment outcomes in patients with advanced-stage *BRAF*-mutated melanoma. Dramatic improvement in the treatment of patients with advanced-stage melanoma has occurred with the addition of checkpoint inhibitors. The use of targeted therapy with immunotherapy, either in sequence or in combination, will likely lead to further improvements in overall survival.

It is of paramount importance that a patient’s *BRAF* mutational status is promptly and accurately determined at the time of initial diagnosis, because it is currently the only reliable predictive biomarker that can influence the treatment of advanced melanoma. VE1 immunostaining for the detection of BRAF-mutated protein is a quick and inexpensive test that can be combined with other melanoma markers. However, since the only mutation that can be detected with the VE1 antibody by IHC is the most common V600E mutant, the presence or absence of other mutations is not determined by this diagnostic test; therefore, evaluation using DNA-based methods, such as the FDA-approved companion diagnostic tests, is still needed. Newer technologies are increasingly being used and refined (eg, evaluation of ctDNA from ‘liquid biopsy’ samples) and will help to address the shortcomings of tests that have been used to date.^72, 73, 76, 77, 81^ Furthermore, the molecular profile of a tumor has been shown to change over time, and inter- and intratumoral heterogeneity can make interpretation of test results challenging. Therefore, it is recommended that repeat testing of the putative primary tumor and recent metastatic sites be performed. Much progress has been made in tests for ctDNA, and this technology holds promise as a tool for early on-treatment monitoring as well as monitoring over time in the context of acquired resistance. It is expected that in this age of precision medicine, the future of *BRAF* biomarker testing will see more techniques move out of the research space and into the clinical practice setting.

## Figures and Tables

**Table 1:** Incidence of common *BRAF* mutations based on the COSMIC database^26, 27^

BRAF *mutation*	*Mutation at codon 600 of* BRAF *gene*	*Incidence in* BRAF-*mutant melanoma, %*
*Common* BRAF *mutations*		
V600E	Valine → glutamic acid	84.6
V600K	Valine → lysine	7.7
		
*Other* BRAF *mutations*		
V600R	Valine → arginine	1
V600M	Valine → leucine	0.3
V600D	Valine → aspartic acid	0.1
Non-V600 mutations (eg, K601E, D594N)	—	<1

**Table 2:** Comparison of methods currently used for *BRAF* mutational analysis

	*THxID-BRAF kit*	*cobas 4800 BRAF V600 mutation test*	*Sanger*	*HRM*	*Pyrosequencing*	*IHC*
**CDx or LDT**	CDx	CDx	LDT	LDT	LDT	LDT
**Sensitivity, %**	>96 for V600E; >92 for V600K	~97 for V600E; 66–70 for V600K	92–98	98–100	>98	85–100
**Specificity, %**	100	>98	100	98–100	90–100	98–100
**Limit of detection, %**	5 for V600E, V600K	5–7 for V600E; >35 for V600K	6.6	6.6	5	5
**Mutations detected**	Approved for V600E, V600K	Approved for V600E only	99% of all detectable mutations	99% of all detectable mutations	Assay optimized for V600 mutations is available	VE1 antibody specific for V600E
**Sample**	Tumor-derived DNA	Tumor-derived DNA	Tumor-derived DNA	Tumor-derived DNA	Tumor-derived DNA	Tissue
**Cost**	Medium	Medium	Medium	Low	High	Low

Abbreviations: CDx, companion diagnostic; HRM, high-resolution melt; IHC, immunohistochemistry; LDT, laboratory-developed test.
